# Production of bioactive recombinant human myeloid‐derived growth factor in *Escherichia coli* and its mechanism on vascular endothelial cell proliferation

**DOI:** 10.1111/jcmm.14602

**Published:** 2019-11-22

**Authors:** Longwei Zhao, Shuang Feng, Shen Wang, Miaojuan Fan, Wei Jin, Xianjing Li, Chen Wang, Yong Yang

**Affiliations:** ^1^ School of Life Science and Technology China Pharmaceutical University Nanjing China; ^2^ Center for New Drug Safety Evaluation and Research China Pharmaceutical University Nanjing China; ^3^ School of Pharmaceutical Sciences & Center for Structural Biology Wenzhou Medical University Wenzhou Zhejiang China

**Keywords:** *E coli* expression system, *human coronary artery endothelial cells*, mitogen activity, recombinant human MYDGF, signal mechanism

## Abstract

Myeloid‐derived growth factor (MYDGF) is a novel protein secreted by bone marrow cells that features important physiological functions. In recent years, MYDGF has gained considerable interest due to their extensive beneficial effect on cardiac repair and protects cardiomyocytes from cell death. However, its precise molecular mechanisms have not been well elucidated. The purpose of this study was to produce sufficient amount of biologically active recombinant human (rh) MYDGF more economically and effectively by using in vitro molecular cloning techniques to study its clinical application. The prokaryotic expression system of *Escherichia coli* was established for the preparation of rhMYDGF. Finally, a large amount of high biologically active and purified form of recombinant protein was obtained. Moreover, we investigated the potential mechanism of rhMYDGF‐mediated proliferation and survival in *human coronary artery endothelial cells* (HCAECs). Mechanistically, the results suggested that MAPK/STAT3 and the cyclin D1 signalling pathways are indispensable for rhMYDGF‐mediated HCAEC proliferation and survival. Therefore, this study successfully established a preparation protocol for biologically active rhMYDGF and it may be a most economical way to produce high‐quality active rhMYDGF for future clinical application.

## 1 INTRODUCTION

According to the statistics of the World Health Organization, acute myocardial infarction (MI) has become one of the major diseases that threaten people's health, and several millions of patients lose their lives due to MI each year.^1^ Therefore, appropriate measures must be taken to protect patients from this serious illness. Although myocardial ischaemia has been effectively alleviated by rapid reperfusion of infarct‐related arteries at present, large loss of cardiomyocytes still occurs during this process.^2^, ^3^, ^4^ Furthermore, it is very difficult for adult heart to self‐regenerate myocardial tissue after MI.^5^, ^6^, ^7^ Therefore, there is an urgent need to identify new therapeutic drugs, develop more effective treatment options for myocardial tissue regeneration and effectively promote the healing of the patient after ischaemia‐reperfusion.

In recent years, the application of autologous bone marrow cells (BMCs) for intracoronary infusion was recommended as effective therapeutic option for improving cardiac tissue regeneration quickly after acute MI.^8^, ^9^, ^10^ Unfortunately, clinical trial results of BMC treatment with intracoronary infusion are controversial due to the uncertain efficacy of autologous cell variable products.^11^ Notably, recent reports have revealed a protein encoded by open reading frame 10 on chromosome 19 (C19orf10) and named as myeloid‐derived growth factor (MYDGF), which is mainly secreted by bone marrow–derived monocytes and murine fibroblasts during adipocyte differentiation.^12^ Moreover, this protein has been shown to be capable of promoting cardiac recovery after MI.^13^, ^14^, ^15^ Besides, subsequent studies showed that MYDGF has a significant therapeutic effect on repairing myocardial tissue compared to BMC infusion. More importantly, recent studies have reported that MYDGF not only reduces ischaemia‐reperfusion injury in vivo, but also attenuates the death of cardiomyocytes from serum starvation, promotes the hyperplasia of human coronary endothelial cells (HCAECs) and improves their ability to form closed tubes.^13^ In addition, a study showed promising results about MYDGF to promote the repair of myocardial tissue after reperfusion injury by reducing the scar size.^13^ Therefore, MYDGF as a novel growth factor may alleviate acute MI and exhibit huge clinical treatment potential in the future. However, the problem of high‐efficiency production of bioactive recombinant human MYDGF is still unsolved, which may seriously limit the application of MYDGF in fundamental research and clinical treatment.

Taking into account this background, the present study aimed to develop an economical method to obtain large amounts of bioactive rhMYDGF. Subsequently, we tested the bioactivity and potential mechanism of rhMYDGF in HCAECs. Given that the MAPK/STAT3/cyclin D1 (encoded by the human CCND1) and PI3K signalling pathways are responsible for cell proliferation, differentiation and survival,^16^, ^17^, ^18^, ^19^, ^20^ we then explained the role of MAPK/STAT3, cyclin D1 and PI3K/AKT signalling pathways in rhMYDGF‐mediated HCAEC proliferation. Taken together, this study demonstrates a more economical method to produce high biologically active form of rhMYDGF, and that would be beneficial for the clinical transformation of MYDGF as a promising therapeutic molecule for the future treatment of patients with ischaemia‐reperfusion injury.

## 2 MATERIALS AND METHODS

### 2.1 Construction of rhMYDGF expression vector

The mRNA sequence encoding the amino acid sequence of human MYDGF was obtained from NCBI database (GenBank accession number of human MYDGF is NM_019107.3) and cloned into the pET31b (Jiancheng Company) enzymatically. NdeI and XhoI (TaKaRa) restriction enzymes were used to produce the pET31b‐rhMYDGF constructor. Subsequently, the recombinant plasmid was transfected into the amplification competent cell of *E coli* DH‐5α (Solarbio Company). Finally, the restriction enzyme analysis and the sequencing methods were used to identify whether the correct cDNA fragments inserted in the vector.

### 2.2 Expression and purification of rhMYDGF

The constructed recombinant plasmid of pET31rb‐rhMYDGF was transformed into BL21 (DE3) pLysS *E coli* cells. The monoclonal strains were selected on agarose gel plates containing 100 μg/mL ampicillin resistances to screen strains with high levels of plasmid expression. The bacterium was inoculated at 1:100 (vol/vol) in fresh LB medium of 30 mL with the concentration of 100 μg/mL ampicillin, and cultured at 37°C in an incubator at 160 rpm until A_600_ reached 0.8 to 1.2, about 8 ~ 10 hours. Next day, the above medium was inoculated at 1:50 (vol/vol) in fresh LB medium of 800 mL containing 100 μg/mL ampicillin. The culture was incubated at 37°C and 180 rpm for 3‐4 hours until A_600_ reached 1.0 to 1.2. IPTG (Generay Biotech Company) was added into the LB medium and dominated a final concentration of 0.5 mmol/L for induction. Then, the temperature was adjusted to 20°C and incubation was continued at 180 rpm for 20 hours. Finally, the cultured bacterial cells were collected by centrifugation at 44400*g* for 10 minutes at 4°C, and the wet cells were labelled and stored at −80°C. The bacterial cells were dissolved in the lysate buffer at 1:40 ratio (wt/vol), mixed and subjected to three rounds (40%, 50% and 55% amplitude) of sonication for 5 minutes each with 5‐seconds interval at 4°C. After equilibrating 3‐5 column volumes of the nickel chelate chromatography column with the equilibration buffer, the supernatants containing soluble proteins were pumped into the nickel column. After the protein with the histidine tag is bound to the column, the column is again equilibrated with buffer for 3‐5 column volumes. Then, gradient elution was performed with different concentrations of nickel column eluent (containing 50, 100 150, 200 and 300 mmol/L imidazole), and the gradient elution peaks were collected. Next, the eluted fractions from the Ni‐NTA column were further purified by gel filtration chromatography (the loading buffer containing 25 mmol/L HEPES, 1 mol/L NaCI and pH: 7.5). Finally, the limulus reagent was used to detect endotoxin produced by *E coli*. The sensitivity of limulus reagent was 0.25 EU/mL by gel semi‐quantitative experiment (Cat# G050250; XIAMEN). All purification steps were performed at 4°C, and the purified rhMYDGF protein was stored at −80°C.

### 2.3 Identification of rhMYDGF purity

The above purified recombinant protein of rhMYDGF needs further identification of its purity by HPLC analysis. Therefore, the Agilent LC1260 high‐performance liquid chromatography and C18 reversed‐phase column (column size 4.6 × 150 mm, particle size 5 μm, pore size 100 μm) were selected for further protein purity identification. Subsequently, recombinant protein sample was bound to the C18 column, firstly. Then, the HPLC protocol was adopted in Agilent 1260 infinity equipped with C18 column according to the protocol provided. In general, the flow phase A was aqueous buffer solution containing 0.1% TFA/H_2_O (V/V) used as the optimum mobile phase. Finally, the target protein was eluted by flow phase B solution with a linear gradient of 0‐90% acetonitrile and at a flow rate of 1.0 mL/min in the presence of 0.1% trifluoroacetic acid (column temperature: 30°C and injection volume: 100 μL). Simultaneously, the absorbance was mainly detected at 280 nm. Elution method was shown in Table S1. Additionally, the purity, degradation rate and polymer of rhMYDGF sample were measured by CE‐SDS analysis as described previously.^21^

### 2.4 Mass spectrometry analysis

The mass spectra of purified recombinant proteins were measured by using the Applied Biosystems Voyager System DEPRO MALDI‐TOF mass spectrometer (Carlsbad) with a nitrogen laser. The matrix solution is a saturated solution consisting of R‐cyano‐4‐hydroxycinnamic acid and acetonitrile and water with volume fraction of 0.1% trifluoroacetic acid, where the ratio of acetonitrile to water with the fraction of 0.1% trifluoroacetic acid is 50:50. Subsequently, the purified rhMYDGF and the above matrix solution were mixed by 1:1 ratio. Then, 1 mL of above mixture was spotted onto a 100‐well sample plate. MS detection was carried out in the range of 700‐600 000 Da under positive ionization mode. The operational mass spectrometric parameters included the following: the accelerating voltage, 20 kV and extraction delay time, 350 ns. Additionally, detection was carried with the 2‐100 kD under linear conditions: accelerating voltage, 25 kV and the extraction delay time, 750 ns.

### 2.5 Western blot analysis

Western blot analysis was applied to identify rhMYDGF, phosphorylation‐MAPK1/3, total‐MAPK1/3, phosphorylation‐STAT3 (S727), phosphorylation‐STAT3 (Y705), total‐STAT3, cyclin D1, phosphorylation‐AKT (T308), phosphorylation‐AKT (S473), total‐AKT and GAPDH. The specific steps are as follows: samples were separated on SDS‐PAGE. After electrophoresis, proteins were transferred to a polyvinylidene difluoride membrane. The membranes were blocked with 5% (wt/vol) skim milk in TBST (0.05% (vol/vol) Tween‐20) for 1 hours at room temperature and then probed with human anti‐rhMYDGF (R&D, Cat# AF1147), mouse anti‐phospho‐MAPK1/3 (Y204/T202, Cat# 4370), rabbit anti‐phospho‐STAT3 (S727, Cat# 9145), rabbit anti‐phospho‐STAT3 (Y705, Cat# 9145), rabbit‐phospho‐AKT (T308, Cat# 13 038), rabbit‐phospho‐AKT (Ser473, Cat# 4060), rabbit anti‐AKT (Cat# 4685), mouse anti‐MAPK1/3 (Cat# 5013), rabbit anti‐STAT3 (Cat# 12 640), mouse anti‐cyclin D1 (Abcam, Cat# ab16663) or goat anti‐GAPDH (Cell Signaling Technology, Cat# 2118) monoclonal antibodies (1:1000) overnight at 4°C. After washing with TBST, the membranes were incubated with peroxidase‐conjugated rabbit antimouse or goat anti‐rabbit secondary antibodies for 90 minutes (1:5000). After three times of additional washing with TBST, the immunoblot strips were detected by using an enhanced chemiluminescence kit. In some experiments, cells were pre‐treated with 20 μmol/L PD98059, 1 nmol/L Stattic or LY294002 (20 μmol/L) for 1 hour. Then, 100 ng/mL activated MYDGF was added into the culture and cells were harvested at different time‐points [the BCA Protein Assay Kit (Cat# P0011, Beyotime) was used to measure the concentrations of MYDGF]. The amount of immunoreactive protein was analysed using ImageJ software version 1.38e (NIH, Bethesda) and normalized against their respective controls.

### 2.6 Cells proliferation analysis

The HCAECs were purchased from ATCC (ATCC^®^ PCS‐100‐020™, primary cells) and cultured in RPMI 1640 supplemented with 10% (vol/vol) FBS and 1% (vol/vol) streptomycin. Firstly, HCAECs were maintained at a density of 5 × 10^6^ in 10 cm dish for three passages at least before experiment (one passage ever 3 days). Then, HCAECs were seeded in 96‐well plates at 3000 cells/well and cultured for 24 hours. Then, HCAECs were starved for 24 hours in serum‐free RPMI 1640 medium and stimulated by the purified rhMYDGF. VEGF and PBS were used as positive and negative control. In addition, HCAECs were stimulated with different concentrations (0, 0.39, 1.56, 6.25, 25 and 100 ng/mL) of rhMYDGF and VEGF for 48 hours (the specific inhibitors of MAPK1/2, STAT3 and PI3K were added in advance). Following treatment, MTT and CCK8 (Beyotime Biotechnology) were added to the 96‐well plates and further incubated for another 4 hours. The absorbance of each well was detected at 490 nm and 450 nm by an immunosorbent assay plate reader (Spectra Max M2, Molecular Devices). In addition, cell proliferation was also evaluated by BrdU incorporation immunoassay (Roche). We inoculated HCAECs in T75 flasks and cultured with EGM‐2 medium (Lonza) containing 10% FBS. Subsequently, we replaced EGM‐2 with MCDB 131 medium (Life Technologies) supplemented with 2% FBS for functional assays. Finally, a colorimetric BrdU incorporation immunoassay (Roche) was applied to detect cell proliferation in 96‐well plates.

### 2.7 Flow cytometry assay

The HCAECs were cultured as described above. Then, the cells were harvested and analysed by flow cytometry. For cell cycle analysis, HCAECs were harvested and resuspended at a density of 1 × 10^5^ cells/mL in 1× cold PBS. After treatment with rhMYDGF in the absence or presence of PD98059 (inhibitor of MAPK, 20 μmol/L) or Stattic (inhibitor of STAT3, 1 nmol/L), 4 mL cold 95% ethanol was then added to each sample to fix the cells for 12‐24 hours. Then, cell cycle analysis was performed with Cell Cycle Assay Kit (FMS‐CCC01, FCMACS) and analysed with flow cytometry according to the manufacturer's instructions. For apoptosis analysis, cells were incubated with rhMYDGF for 24 hours. Then, cells were added with H_2_O_2_ (200 μmol/L) for another 24 hours in the absence or presence of PD98059 and static, and 1 × 10^5^ HCAECs were then resuspended in 1× binding buffer and stained with Annexin V and 7‐AAD following the manufacturer's instructions of apoptosis assay kit (FMSAVPE‐100; FCMACS). Cells were analysed using flow cytometry to detect early and late apoptosis of cells. All experiments were performed in triplicate.

## 3 RESULTS

### 3.1 Construction of recombinant plasmid, expression and purification

**Figure 1:**
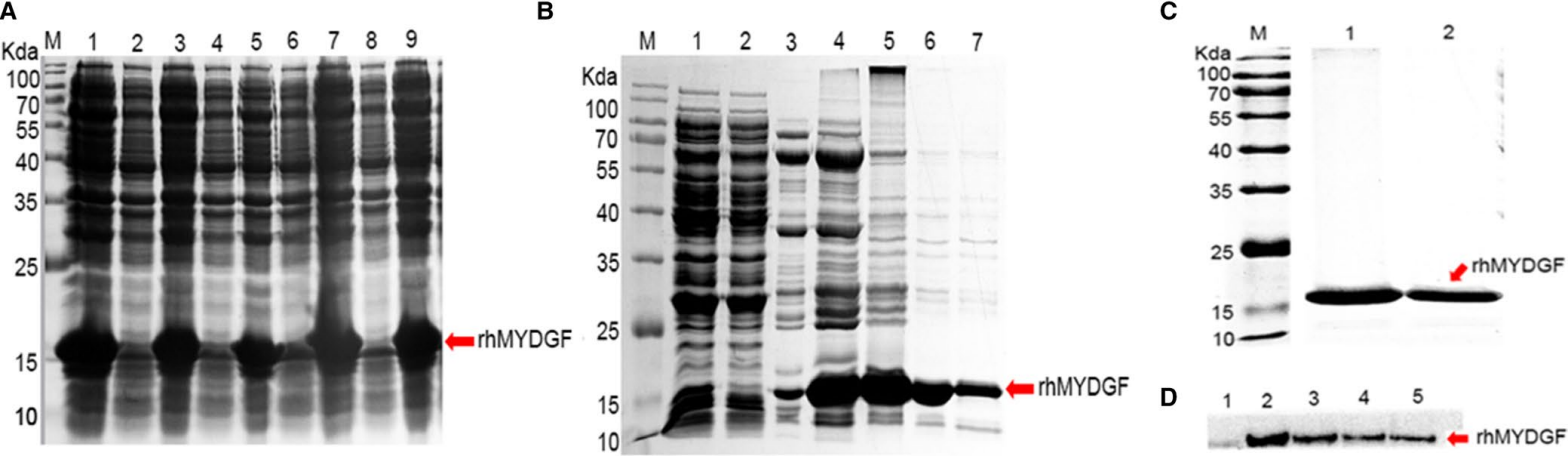
Expression and purification of the recombinant human MYDGF and target protein. A, Schematic representation of expression vector pET31b‐rhMYDGF. Lane M molecular weight standards: Lanes 2, 4, 6 and 8 represent the uninduced BL21 (DE3)/pET31b‐rhMYDGF in different batches: Lanes 1, 3, 5, 7 and 9 represent the induced BL21 (DE3)/pET31b‐rhMYDGF in different batches. B, Schematic representation of nickel column eluted sample, Lane M: molecular weight marker. Lane 1: sample; Lane 2: unbound sample; Lanes 3, 4, 5, 6 and 7: eluted with 50, 100, 150, 200 and 300 mmol/L imidazole, respectively. C, Schematic representation of gel filtration chromatography eluted sample, Lane M: molecular weight marker: Lane 1 and 2: target protein sample eluted after gel filtration chromatography. D, Expression of the His‐rhMYDGF protein analysed by Western blotting. Lane 1, uninduced; Lanes 2, 3, 4 and 5: after induction for 20, 16, 12 and 8 h at 20°C

We first constructed the recombinant plasmid by inserting the DNA sequence encoding rhMYDGF into vector (Figures S1A and S2A). Then, the recombinant plasmid was validated by restriction enzyme digestion, producing two bands: one at 5000∼6000 bp is corresponding to pET31b and the other at 400∼500 bp is representing the DNA fragment of rhMYDGF (Figure S1B), suggesting the inserted DNA sequence was a correct fragment of encoding MYDGF. Thereafter, the pET31b‐rhMYDGF was transfected into expressed competent cell of *E coli* BL21(DE3) pLysS for expressing the recombinant protein and our results indicated that rhMYDGF was mainly expressed in the supernatant (Figure 1A). Then, the Western blot further demonstrated the protein band at ~17 kD presence of an immunoreaction with MYDGF antibody (Figure 1D). The above protein supernatant was first bound to the nickel affinity column and then eluted by the different concentrations of imidazole (50‐300 mmol/L), and the SDS‐PAGE analysis showed that we obtained the purer recombinant protein at the concentration of 200‐300 mmol/L imidazole (Figure 1B). Finally, the gel filtration column was applied to obtain high purity protein with the purity >95% (Figure 1C) and the concentration of the final protein solution endotoxin is between 5 and 10 EU/mg. Additionally, we also evaluated the amount of the polymer and the purity of the sample by CE‐SDS analysis, and the results showed that the ratio of main peak was 96.13%, 0.84% of degradant, and the ratio of polymer was 3.02% (Figure S3). We also inferred that there may be no disulphide in our final product by the analysis of SDS‐PAGE in the presence or absence of DTT and mass spectrometry (Figures S4 and S5).

### 3.2 Mass spectrometry identification of rhMYDGF

**Figure 2:**
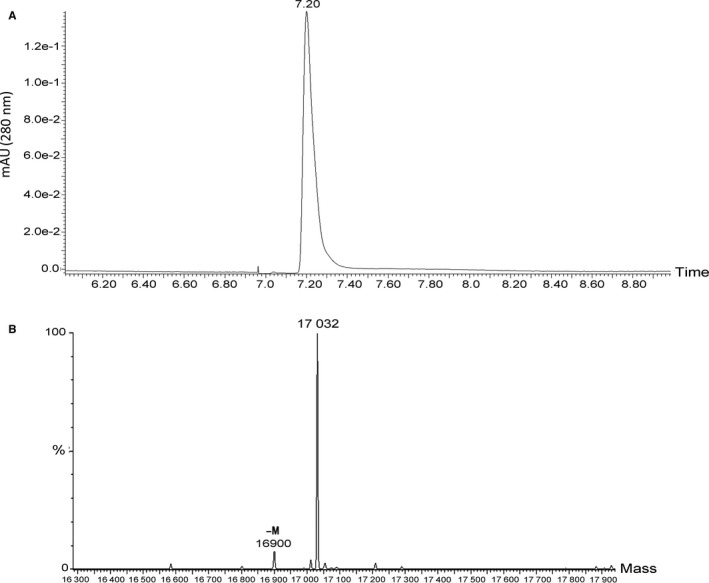
Identification of rhMYDGF by HPLC and mass spectrometry. A, HPLC analysis of the purified rhMYDGF. The purity of rhMYDGF eluted from a heparin affinity column was further evaluated by HPLC analysis using a C18 column. As seen from the chromatogram, the y‐axis indicates the absorbance (280 nm, AU), while the x‐axis showed elution time (min). The main peak was observed at 7.20 min. The purity of purified rhMYDGF was more than 95%. B, Mass spectrometry analysis of the rhMYDGF. The peptide mixture of rhMYDGF was separated by the C4 reversed‐phase column to identify its molecular weight. The y‐axis indicates the relative abundance. While the x‐axis showed molecular weight, the main peak was observed at 17 032 Da

It is well known that high‐performance liquid chromatography (HPLC) combined with MALDI‐TOF MS mass spectrometry is usually applied to detect protein molecular weight and amino acid sequence. To further identify the above‐obtained purity protein, the purity protein samples were subjected to HPLC and obtained a single peak at the retention time of 7.20 minutes (Figure 2A). Then, we collected the single peak sample and measured its molecular weight by MALDI‐TOF MS and the amino acid sequence of the purity protein was determined by following the protocol of MS/MS. It is worth noting that the mass spectrometry results showed the molecular weight of the main single peak after reversed‐phase C4 separation was 17 032 Da (17.032 kD; Figure 2B), 0.92 Da from the theoretical molecular weight, and was substantially the same as the theoretical molecular weight of 17 031.08 Da (Figure S2B and C). Additionally, the other small single peak was observed by the result of mass spectrometry with a signal of 16900 Da (16.9 kD), which may be the degradation product of the protein. Moreover, to detect the amino acid sequence of the purity protein, the protein sample first needs to be treated by peptidase to completely dissociate the peptide bonds in the structure, then the thiol group is labelled with IAM, and finally, the sample is digested with trypsin to make it into different peptide segments. Immediately, after the different peptides are processed by mass spectrometry and data analysed, the peptide sequence coverage information of the sample can be obtained. Notably, our results suggested the peptide sequence coverage of this purity protein is 98% (Figure S5), demonstrating most peptide sequences can be verified by secondary fragment information. However, there is an ASR mismatch in the peptide sequences, it may be that the peptide is weakly hydrophobic and cannot be detected at dead time (Figure S5).

### 3.3 Mitogenic activity of rhMYDGF on HCAECs

**Figure 3:**
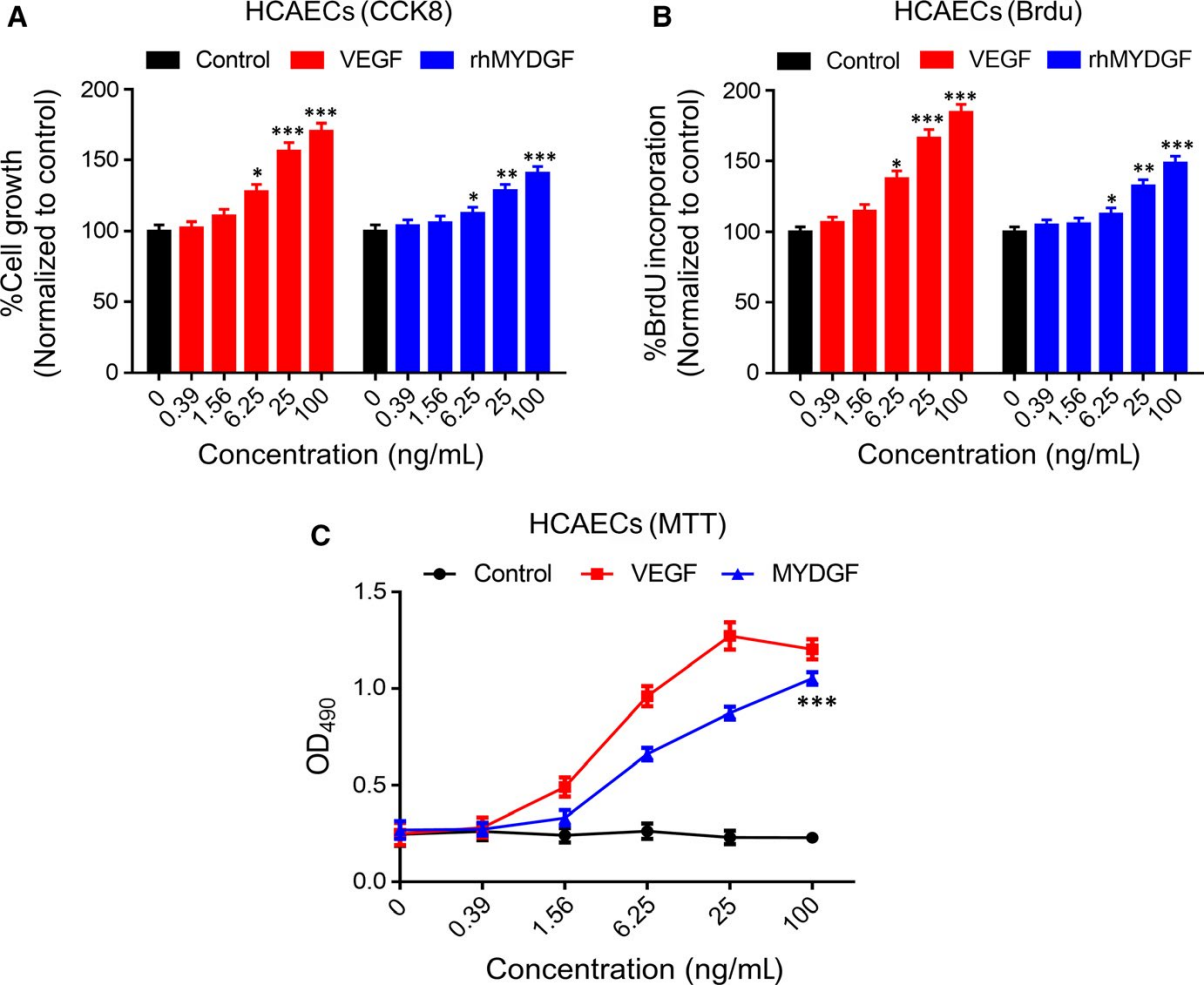
Mitogenic and proliferation activity analysis of different concentrations of rhMYDGF on HCAECs in vitro. A, Cells were plated into 96‐well plates and incubated for 24 h, and then serum‐starved for 24 h. The rhMYDGF and VEGF were added for 48 h, after which CCK8 incorporation was measured, as described in ‘Materials and methods’ section. Five independent experiments were performed. B, BrdU incorporation in HCAECs after stimulation for 16 h with different concentrations of VEGF and rhMYDGF. Five independent experiments were performed. C, Cells were plated into 96‐well plates and incubated for 24 h, and then serum‐starved for 24 h. The rhMYDGF and VEGF were added for 48 h, and cell viability was measured by MTT assay, as described in ‘Materials and methods’ section. *P < .05, **P < .01 and ***P < .001 vs baseline (two independent‐sample *t* test). The number of cells in the control group represents the 100%

To evaluate the biological activity of our recombinant protein, we selected HCAECs to test the proliferative activity of rhMYDGF in vitro. Therefore, MTT, CCK8 and BrdU assays were used to evaluate the effects of purified rhMYDGF on the proliferation of HCAECs in vitro. The data obtained from three independent experiments demonstrated that rhMYDGF significantly promotes the proliferation of HCAECs in a dose‐dependent manner (Figure 3A, 3 and 3), which were indicated by the OD value or the percentage of cell growth (normalized to control). Moreover, as we all known, VEGF is a highly specific pro‐vascular endothelial growth factor with strong ability to promote vascular proliferation.^22^ Interestingly, we observed that the proliferation of HCAECs induced by rhMYDGF was almost similar to that of VEGF treatment, and its EC_50_ was 7.09 ± 0.04 ng/mL. Notably, the results of the three independent experimental assays (CCK8, BrdU and MTT assays) for cell proliferation showed that VEGF group was of high magnitude compared to rhMYDGF treatment, which may be associated with the different ability of VEGF and rhMYDGF to promote cell proliferation, as demonstrated by an upward shift in their dose‐response curve. Overall, the three independent experiments of MTT, CCK8 and BrdU assay showed a similar effect of rhMYDGF in HCAEC proliferation and mitogenic activity in vitro.

### 3.4 MAPK1/3/STAT3, cyclin D1 and PI3K/AKT signal activation in HCAECs were regulated by exogenous rhMYDGF

**Figure 4:**
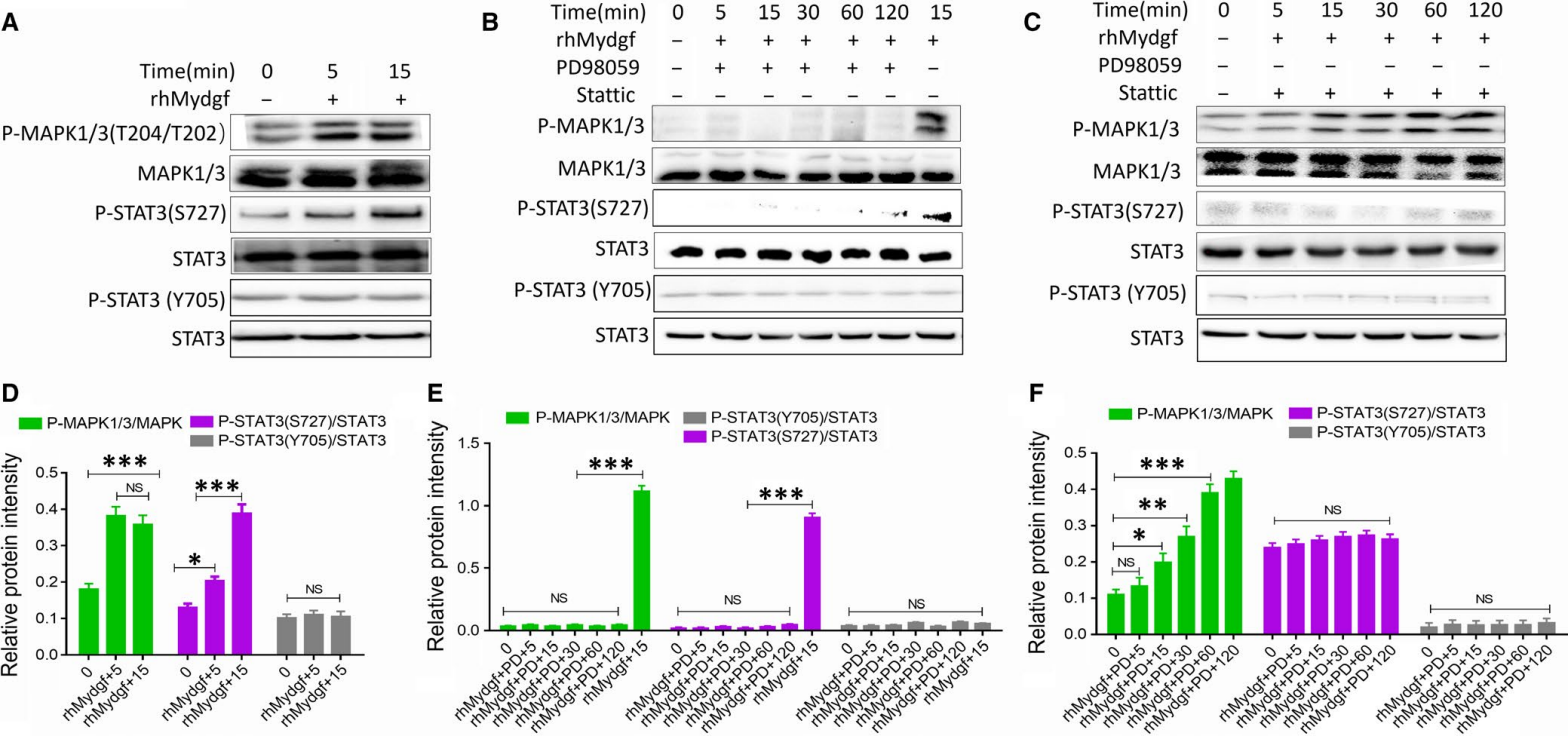
rhMYDGF promotes HCAEC proliferation and is unstimulated or stimulated with rhMYDGF (100 ng/mL unless otherwise indicated), PD98059 (PD, 20 µmol/L) or Stattic (1 nmol/L), as indicated. A, Representative immunoblots showing phosphor (p)‐MAPK1/3 (Y204/T202), MAPK1/3, p‐STAT3 (S727) and p‐STAT3 (Y705) expression in HCAECs. B, Representative immunoblots showing p‐MAPK1/3, MAPK1/3, p‐STAT3 (S727) and p‐STAT3 (Y705) expression in HCAECs in the presence of MAPK1/3 inhibitor (PD98059). C, Representative immunoblots showing p‐MAPK1/3, MAPK1/3, p‐STAT3 (S727) and p‐STAT3 (Y705) expression in HCAECs in the presence of STAT3 inhibitor (Stattic). D‐F, Western blot analysis was performed to examine the protein levels of p‐MAPK1/3 and p‐STAT3 by ImageJ. *P < .05, **P < .01 and ***P < .001 vs baseline (two independent‐sample *t* test)

**Figure 5:**
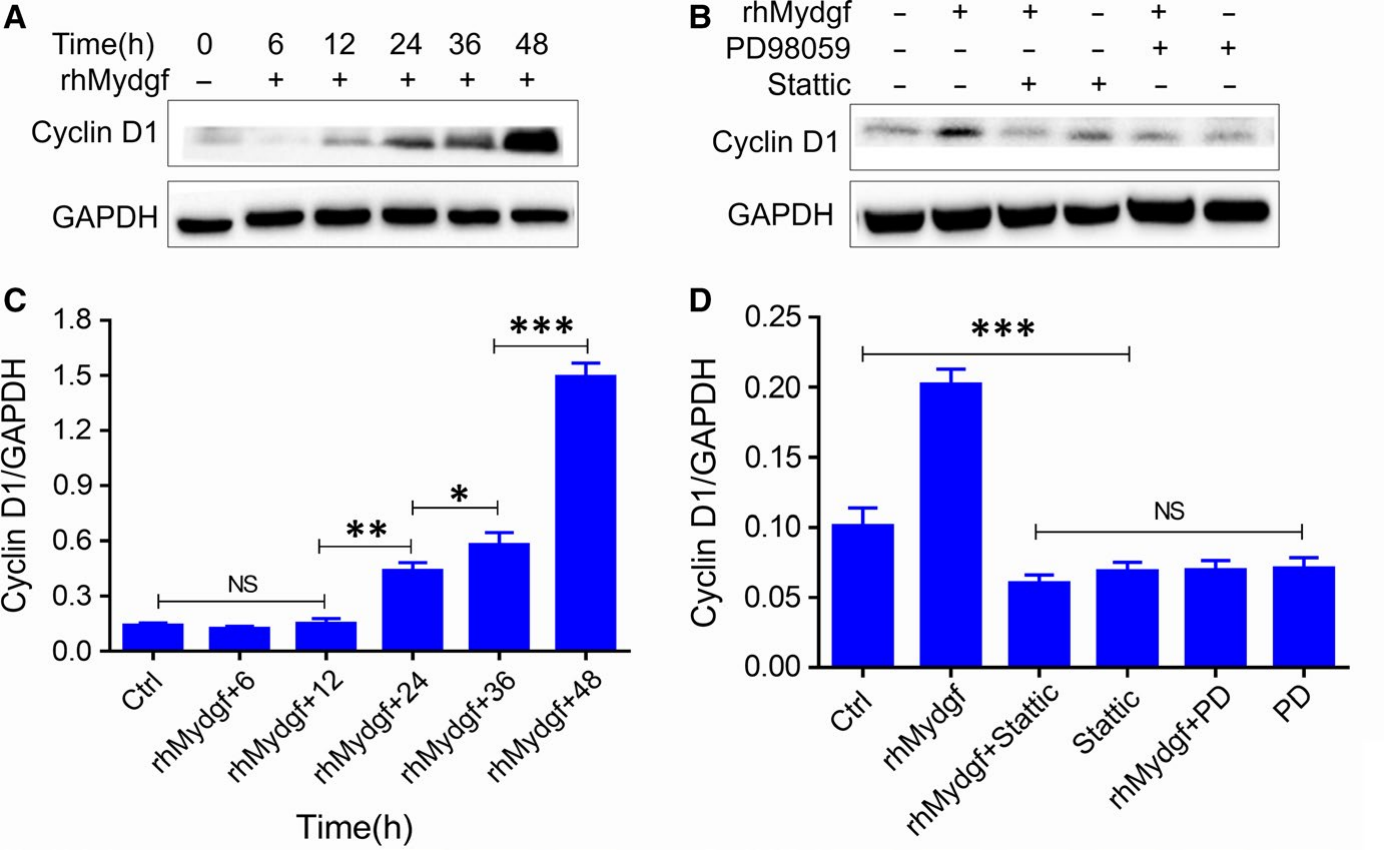
rhMYDGF promotes HCAEC proliferation. HCAECs were stimulated with rhMYDGF (100 ng/mL unless otherwise indicated) in the absence or presence of PD98059 (PD, 20 µmol/L) or Stattic (1 nmol/L), as indicated. A, Representative immunoblots showing cyclin D1 and GAPDH expression in HCAECs. B, Representative immunoblots showing cyclin D1 and GAPDH expression in HCAECs stimulated for 12 h. C‐D, Western blot analysis was performed to examine the protein levels of cyclin D1. *P < .05, **P < .01 and ***P < .001 vs baseline (two independent‐sample *t* test)

It is well known that MAPK/STAT3 and PI3K signalling pathways are important for cell growth and survival.^20^, ^23^, ^24^ Therefore, we aimed to determine whether the rhMYDGF‐mediated HCAEC proliferation is associated with MAPK/STAT3 or PI3K signalling pathway. Notably, consistent with previous report, when HCAECs were treated with 100 ng/mL of rhMYDGF protein in vitro for 0, 5 and 15 minutes, respectively, the phosphorylation of MAPK1/3 and STAT3 on S727 was significantly enhanced with the time extending, while that did not occur at vehicle treatment (Figure S6), suggesting the effect of rhMYDGF on HCAEC mitosis was closely related to the activation of MAPK1/3/STAT3 (S727), whereas rhMYDGF did not increase the phosphorylation of STAT3 on Y705 (Figure 4A and 4), which is required for STAT3 dimerization and nuclear translocation.^25^ Moreover, the results also demonstrated the expression of cyclin D1 protein was gradually increased with the time extending (0, 6, 12, 24, 36 and 48 hours), suggesting that cyclin D1 also participated in the progression of cell proliferation (Figure 5A and 5).^26^ Notably, rhMYDGF also could activate the PI3K/AKT signalling pathway by increasing the phosphorylation of AKT on T308 and S473 and the effect was abolished in the presence of LY294002 (the specific inhibitor of PI3K; Figure S7A and B).

### 3.5 MAPK/STAT3 and cyclin D1 signalling pathways are obligatory for the mitogenic activity of rhMYDGF on HCAECs

**Figure 6:**
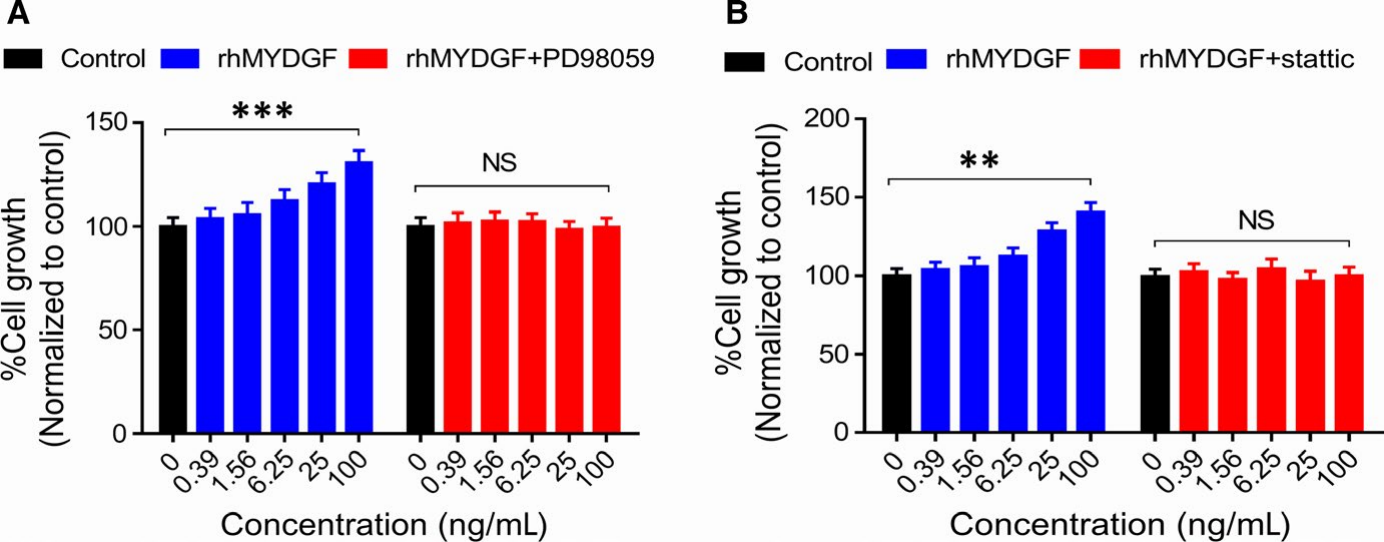
Mitogenic and proliferation activity analyses of HCAECs with different concentrations of rhMYDGF in the absence or presence of MAPK1/3 and STAT3‐specific inhibitors PD98059 (20 μmol/L) and Stattic (1 nmol/L) in vitro.A,HCAECs were treated with increasing concentrations of rhMYDGF in the absence or presence of MAPK1/3‐specific inhibitors PD98059. B, HCAECs were treated with increasing concentrations of rhMYDGF in the absence or presence of STAT3‐specific inhibitors Stattic. *P < .05, **P < .01 and ***P < .001 vs control (two independent‐sample *t* test)

The above results showed that rhMYDGF promotes HCAEC proliferation, which was associated with the activation of MAPK/STAT3 and cyclin D1 signalling pathways (Figure 5A and 5). Next, we wanted to investigate whether this signalling pathway is indispensable for rhMYDGF‐mediated HCAEC proliferation. Therefore, the specific inhibitors of MAPK kinase 1 (PD98059) and STAT3 (Stattic) were applied to further explore the molecular mechanism underlying rhMYDGF‐induced cell proliferation.^27^, ^28^, ^29^, ^30^ To test this, rhMYDGF was added to each group, and then, the total proteins were harvested at indicated time‐points (0, 5, 15, 30, 60 and 120 minutes). Western blotting analysis demonstrated that phosphorylation of MAPK1/3 and STAT3 induced by rhMYDGF was dramatically decreased by PD98059 (Figure 4B), while that did not occur in the presence of static (specific inhibitor of STAT3; Figure 4C). In addition, results also demonstrated that the expression of cyclin D1 could be prevented by treatment with PD98059 or Stattic (Figure 5B and 5). Notably, the effect of endothelial cell proliferation mediated by rhMYDGF was abrogated after pre‐treating HCAECs with PD98059 and static (Figure 6A and 6), demonstrating the expression of cyclin D1 is required for HCAEC proliferation. Overall, the above data demonstrated that MAPK/STAT3 signalling pathway and cyclin D1 are indispensable for rhMYDGF to promote HCAEC growth, while the expression of cyclin D1 was regulated by MAPK/STAT3 signalling pathway. However, the PI3K inhibitor of LY294002 did not abolish the effect of rhMYDGF‐mediated proliferation on HCAECs, conforming the PI3K/AKT signalling pathway did not participate in rhMYDGF‐mediated HCAEC proliferation (Figure S7C).

### 3.6 The effect of rhMYDGF on Cell Cycle and Apoptosis

**Figure 7:**
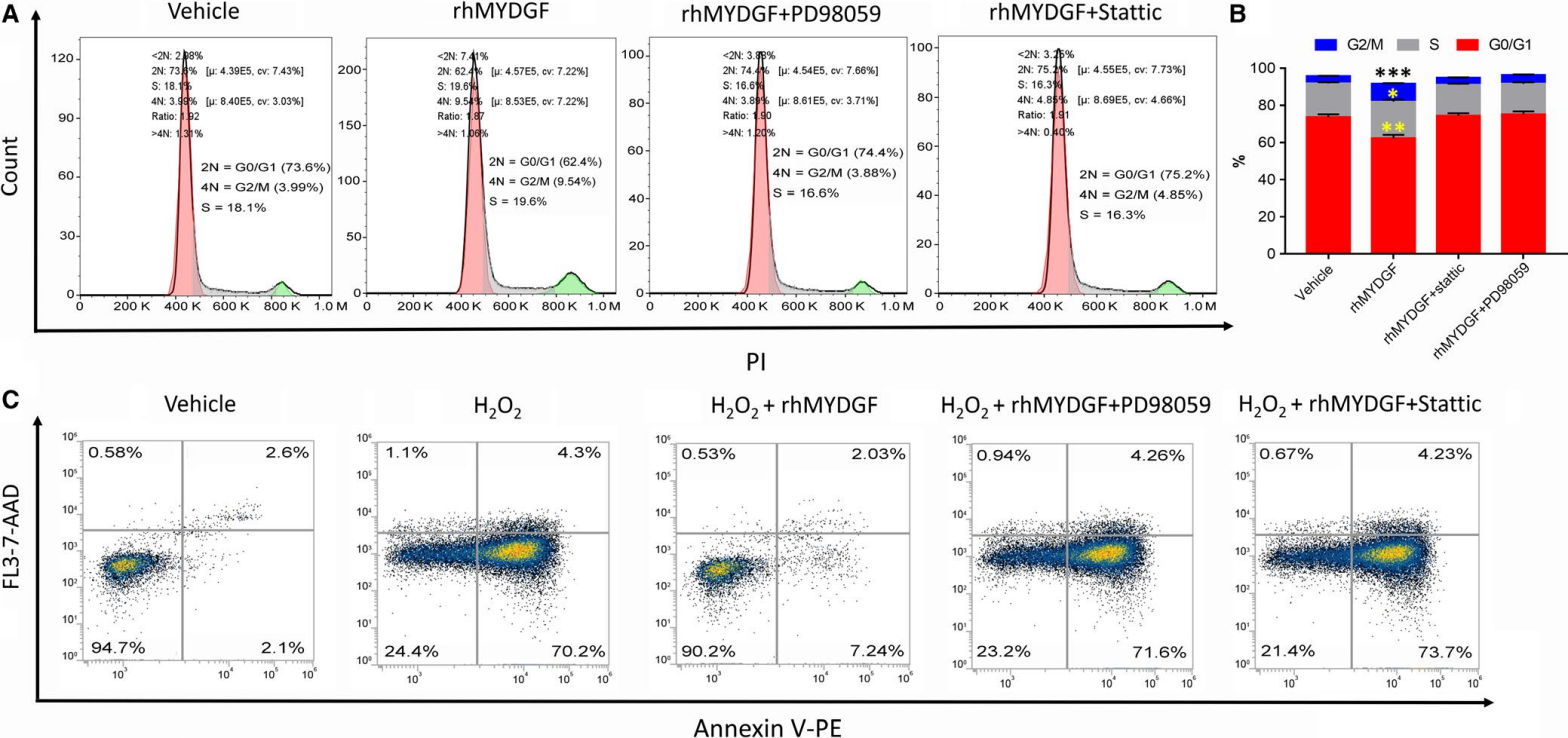
The effect of rhMYDGF on cells cycle regulation and apoptosis. A, Flow cytometry analysis showing changes in cell cycle phases after treatment with rhMYDGF (100 ng/mL) in the absence or presence of PD98059 and Stattic. B, Proportion of each phase in the cell cycle of different groups, *P < .05, **P < .01 and ***P < .001 vs vehicle. C, Flow cytometry analysis showing that pre‐incubation with rhMYDGF (100 ng/mL) for 24 h inhibited H_2_O_2_ (200 μmol/L)‐induced apoptosis in HCAECs in the presence or absence of PD98059 (20 μmol/L) and Stattic (1 nmol/L). A total of 100 000 cells were analysed in each group. The right upper quadrant indicates the late apoptotic cells; the right lower quadrant indicates the early apoptotic cells, and the left upper quadrant indicates necrotic cells

In order to further confirm the importance of MAPK1/3/STAT3 and cyclin D1 signalling pathways for rhMYDGF‐mediated HCAEC proliferation and survival, we evaluated the effect of rhMYDGF on cell cycle in the absence or presence of PD98059 or static. Interestingly, the results demonstrated that the percentage of cells at S and G2/M phase were increased after treatment with rhMYDGF compared to control and presence of PD98059 or static, suggesting the effect of rhMYDGF on HCAEC proliferation (Figure 7A and 7). Next, to investigate the protective effect of rhMYDGF on cell apoptosis, we pre‐treated the HCAECs with rhMYDGF in the absence or presence of the inhibitors of MAPK and STAT3; then, 200 μmol/L H_2_O_2_ was added into the cell medium for 24 hours. The results showed that pre‐incubation with rhMYDGF could significantly inhibit H_2_O_2_‐induced HCAEC apoptosis, and the above effect was abolished in the presence of PD98059 or Stattic (Figure 7C).

## 4 DISCUSSION

The protein of MYDGF was secreted by a bone marrow–derived stromal cell line and initially named as SF20 or interleukin‐25 (IL‐25) as for its ability to promote lymphocyte proliferation.^31^ However, this activity has not been reproducible, and thus, the function of this protein is currently unknown [provided by RefSeq, Jul 2008]. Therefore, MYDGF could not be equated to SF20 or IL‐25 in vivo. Notably, recent studies demonstrated that MYDGF is secreted from murine fibroblasts during adipocyte differentiation.^12^ In addition, MYDGF is highly expressed in spleen, prostate and lung, whereas it is weakly expressed in the other organs (such as the left ventricle and liver in mice under baseline conditions).^13^ Mortimer Korf‐Klingebiel et al also demonstrated that MYDGF has effective abilities to promote heart repair after MI and treatment with this protein not only avoids the combined effects of intracoronary infusion of autologous bone marrow cells, but also promotes cardiac recovery effectively after MI. Those characteristics would bring the promising lives to patients who suffered from ischaemia‐reperfusion injury. Thus, the protein is suggested to develop into a potential candidate for the treatment of MI and could be applied to the clinical treatment in the future. However, the premise of becoming a clinical drug also requires a large amount of preclinical research. At present, small amount of rhMYDGF for basic research was obtained by HEK‐293T cells or mammalian cell expression system (CHO). However, the larger‐scale production of rhMYDGF by the mammalian cell expression system (CHO) requires high cost. Obviously, the present strategies are not the best choice from the perspective of economic efficiency or cost. Therefore, the basis for the evaluation and development of this protein drug is to establish a simple and economical protein preparation programme, which is also the goal of our current work. Therefore, in this study, we have chosen a relatively more cost‐effective expression system of prokaryotic to produce our target protein.

Previous studies have shown that purification of a target protein should consider the simplicity and efficiency.^32^, ^33^ However, natural proteins are generally naked proteins, which will bring a series of problems for purification and is often difficult to obtain highly pure target proteins. Therefore, the naked proteins are often attached to a tag for purification.^34^ Previously, it has been reported that the C‐terminal fusion of histidine tag does not affect the transcription and translation of bacteria. Moreover, compared with glutathione S‐transferase (GST) tags, the molecular weight of histidine tags is small and difficult to form dimers to affect the activity of the protein.^35^

Therefore, in this study, we chose the pET31b vector containing a His tag to express the target recombinant protein (Figure 1A). Interestingly, we obtained large amount of soluble rhMYDGF proteins by the nickel affinity column and gel filtration chromatography (Figure 1B and 1). Finally, the HPLC and mass spectrometry were applied to further analyse the above collected sample. Collectively, the above results indicated that we had successfully established an efficient and economical strategy for obtaining a large amount of high purity rhMYDGF with low endotoxin (5‐10 EU/mg) and a yield of 216 mg rhMYDGF per 100 g wet cells (Table S2). Subsequently, to identify the biological activity and potential mechanism of the recombinant protein in cell proliferation, the relative experiments were carried out as follows.

Previous studies have reported that the MAPK/STAT3 and PI3K/AKT signalling pathways play the very important role in the promotion of mitogenic and proliferation of cells.^20^, ^23^, ^36^ In this study, the results demonstrated that our obtained protein could promote the mitogenic activity and proliferation of HCAECs, which is similar to VEGF in HCAECs, and the effect of both rhMYDGF and VEGF on cell proliferation was dose‐dependent (Figure 3A, 3 and 3), while the VEGF exhibited a stronger proliferative activity than MYDGF (Figure 3A, 3 and 3), which may be associated with its strong ability to promote vascular proliferation through VEGFR for as a highly specific pro‐vascular endothelial growth factor, which may have potential risk for haemangioma formation in vivo. MYDGF as a paracrine adipocytokines and its specific receptor have been still unknown, and it showed a weak effect on HCAEC proliferation compared to VEGF, which may be more secure in future clinical applications. To explain the mechanism of rhMYDGF that promotes HCAEC proliferation, we conducted a series of experiments in HCAECs including signalling pathway detection, cell cycle and apoptosis in the presence or absence of inhibitors (PD98059 and Stattic). Notably, rhMYDGF could induce the phosphorylation of MAPK and STAT3 on S727, as well as the expression of cyclin D1 in vitro, suggesting MAPK1/3, STAT3 (S727) and cyclin D1 may mediate the effect of rhMYDGF on HCAEC proliferation, which is consistent with the previous studies that STAT3 serine phosphorylation on S727 is important for cellular growth,^25^, ^37^ whereas rhMYDGF did not enhance the phosphorylation of STAT3 on Y705, which is required for STAT3 dimerization and nuclear translocation, and the relative contribution of phospho‐S727 to the regulation of STAT3 tyrosine phosphorylation in vivo is presently unclear.^25^, ^37^

The above results are consistent with the previous report.^13^ In addition, our results suggested that although rhMYDGF can activate PI3K/AKT signalling pathway in the absence of LY294002, it did not involve in rhMYDGF‐mediated HCAEC proliferation (Figure S7), suggesting the PI3K signalling pathway may mediate other biological functions of rhMYDGF except for proliferation in HCAECs and confirming the importance of MAPK/STAT3 and cyclin D1 for rhMYDGF‐induced endothelial cell proliferation. Therefore, to investigate the specific relationship among MAPK1/3, STAT3, expression of cyclin D1 and the effect of rhMYDGF in HCAEC proliferation, MAPK1/3 and STAT3 signalling pathways were specifically inhibited before the rhMYDGF treatment. It is worth noting that STAT3 inhibitor could effectively inhibit rhMYDGF‐induced S727 phosphorylation of STAT3,^27^ but have no effect on MAPK1/3 and MAPK3 phosphorylation (Figure 4C). However, MAPK1 inhibitor could inhibit phosphorylation of MAPK1 and MAPK3 as well as STAT3 on S727 significantly (Figure 4B), indicating that STAT3 is the downstream signal of MAPK. Furthermore, when the HCAECs were treated with different concentrations of rhMYDGF, and co‐incubation with MAPK1/3 and STAT3 specific inhibitors of PD98059 and Stattic in vitro*,* the proliferative capacity of rhMYDGF on HCAECs was missed. In addition, the results of cell cycle and apoptosis also demonstrated the importance of MAPK/STAT3 signalling pathway for rhMYDGF‐mediated HCAEC proliferation and survival (Figure 7). Therefore, the MAPK/STAT3 signalling pathway was indispensable for rhMYDGF to promote cells proliferation and survival. Moreover, it is emphasized that the cyclin D1 gene is closely associated with STAT3 activation.^38^ Notably, rhMYDGF also increased cyclin D1 protein expression with the time extending under a certain dosing. However, when the specific inhibitors of MAPK1/3 and STAT3 were applied to HCAECs prior to rhMYDGF treatment, the protein expression of cyclin D1 was significantly decreased, suggesting the expression of cyclin D1 is regulated by MAPK and STAT3 signalling pathways. In other words, the ability of rhMYDGF to promote cell proliferation depended on the MAPK/STAT3 signalling pathway to up‐regulate the expression of cyclin D1. This mechanism is similar to the conclusion of a previous study, which demonstrated that QKI‐5 could suppress cyclin D1 expression and proliferation of oral squamous cell carcinoma cells via the MAPK signalling pathway.^26^

**Figure 8:**
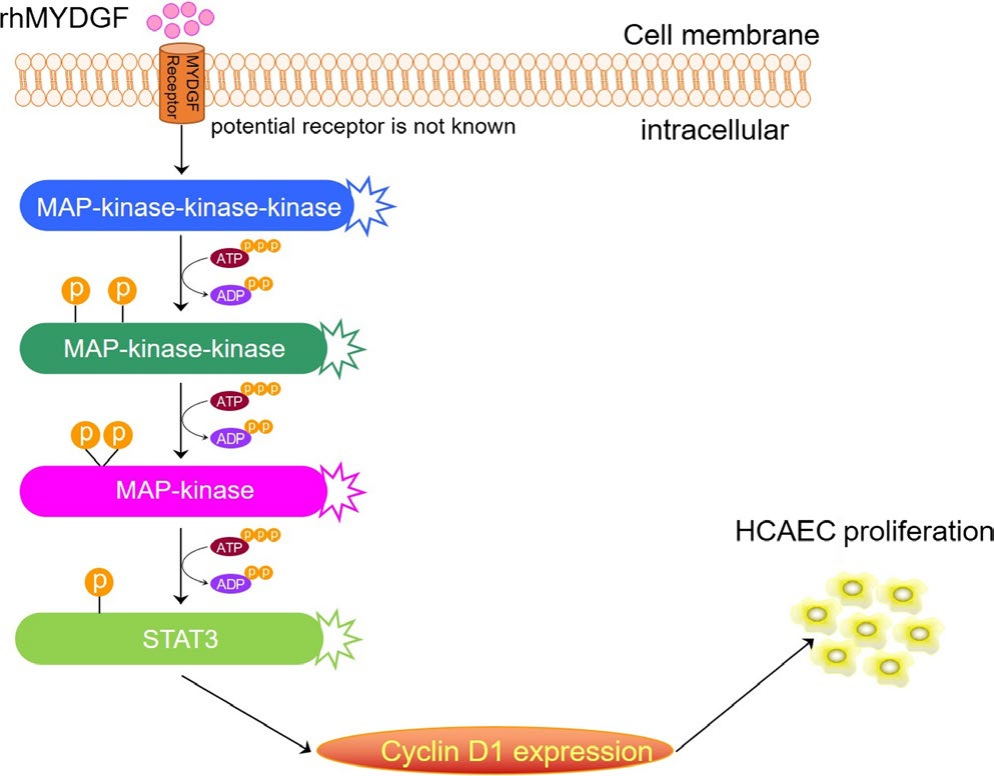
A working model of the potential mechanism of rhMYDGF‐mediated proliferation on HCAECs

In summary, this study provided a simple and efficient purification strategy for producing a bioactive and soluble rhMYDGF recombinant protein with >95% purity. Furthermore, this investigation shows the importance of MAPK/STAT3 and cyclin D1 signalling pathways in rhMYDGF‐mediated proliferation and survival of HCAECs. Moreover, the mechanism of rhMYDGF‐mediated proliferation in cells revealed that the expression of cyclin D1 is directly dependent on the phosphorylation of STAT3 on S727 rather than MAPK, but the phosphorylation of STAT3 is directly regulated by MAPK1/3. Therefore, the MAPK/STAT3 signalling pathway is essential in the regulation of HCAEC proliferation and survival, which is consistent with the previous study.^13^ Our strategy provides a simple method for producing high‐quality active rhMYDGF, making it convenient for clinical application ahead. Regrettably, due to the unclear receptor of MYDGF, the specific mechanism could not be further elaborated. Thus, finding the specific receptor for MYDGF and further elucidating its pharmacological mechanism would be the top priority of our next study (Figure 8).

## CONFLICT OF INTEREST

The authors confirm that there are no conflicts of interest.

## AUTHOR CONTRIBUTIONS

LZ wrote the first draft of the manuscript. L.Z and SW contributed to the conception and design of the research. LZ, SF, MF, WJ and XL contributed to the experiment and analysis of the data. CW and YY contributed to the analysis and interpretation of the data. All authors critically revised the manuscript, agreed to be fully accountable for ensuring the integrity and accuracy of the work, and read and approved the final manuscript.

## Data Availability

All data used for this project are publicly available and accessible online.
