# Supplementary material for: Production of bioactive recombinant human myeloid‐derived growth factor in Escherichia coli and its mechanism on vascular endothelial cell proliferation
Source: J Cell Mol Med. 2019 Nov 22;24(2):1189–99. doi: 10.1111/jcmm.14602 (PMC6991672; doi:10.1111/jcmm.14602)
Supplement: Supplementary file 1 [file JCMM-24-1189-s001.docx]

**Table S1** Liquid phase separation gradient

|  | Time（min） | Flow (ml/min) | A% | B% |
| --- | --- | --- | --- | --- |
| 1 | 0 | 1.0 | 100 | 0 |
| 2 | 8 | 1.0 | 100 | 0 |
| 3 | 68 | 1.0 | 40 | 60 |
| 4 | 72 | 1.0 | 60 | 60 |
| 5 | 75 | 1.0 | 100 | 0 |
| 6 | 80 | 1.0 | 100 | 0 |

The above data was the elution parameter of HPLC analysis.

**Table S2** Summary of the purification of rhMYDGF

|  | Wet cells (g) | Volume (ml) | Protein concentration (mg/ml) | Total protein (mg) | Purity (%) | Yield (mg/100 g wet bacteria) |
| --- | --- | --- | --- | --- | --- | --- |
| Protein solubilization | 100 | 1000 | 0.82 | 820 | 46% | 377.2 |
| Nickel-chelating  column |  | 328 | 0.93 | 305.04 | 80% | 244.032 |
| Gel filtration chromatography column |  | 150 | 1.5 | 225 | 96% | 216 |

The above data was the average of three independent experiments. The protein was quantified by BCA method. The amount of target proteins was estimated by densitometry analysis of the protein band in SDS-PAGE gels. Total protein = protein concentration (mg/mL) × volume (mL). Yield = total protein (mg) × purity (%).
