# Supplementary material for: Production of bioactive recombinant human myeloid‐derived growth factor in Escherichia coli and its mechanism on vascular endothelial cell proliferation
Source: J Cell Mol Med. 2019 Nov 22;24(2):1189–99. doi: 10.1111/jcmm.14602 (PMC6991672; doi:10.1111/jcmm.14602)
Supplement: Supplementary file 2 [file JCMM-24-1189-s002.docx]

**Supporting information**

**Journal:** [Journal of Cellular and Molecular Medicine](https://www.baidu.com/link?url=QNj1P4eoSZKFCJe7KDU44fQK3ByobCQ1Iy_-8_OUxrz3dCGufPhst96_7BOgyCGSReRcTVOru4lFFbqypA3C2w_aSV1kFuiGImteaHsc5AC&wd=&eqid=9ef6fc5100050466000000055cde0f9c).

**Title:** Production of bioactive recombinant human myeloid-derived growth factor in *E. coli* and its mechanism on vascular endothelial cells proliferation

**Authors:** Longwei Zhao^1,2^, Shuang Feng^2^, Shen Wang^1^, Miaojuan Fan^3^, Wei Jin^3^, Xianjing Li^1,2^, Chen Wang^1^* and Yong Yang^1,2^*

^1^ School of Life Science and Technology, China Pharmaceutical University, Nanjing 210009, PR China

^2^ Center for New Drug Safety Evaluation and Research, China Pharmaceutical University, Nanjing 211198, PR China

^3^ School of Pharmaceutical Sciences & Center for Structural Biology, Wenzhou Medical University, Wenzhou, Zhejiang 325035, China

*To whom correspondence should be addressed:

E-mail: [yy@cpu.edu.cn](mailto:yy@cpu.edu.cn)

E-mail: cwang1971@cpu.edu.cn

Phone and Fax: 86-025-86185622

**9 pages**

**7 Figures**

**The following is included as Supporting Information for this paper:**

**Supplementary Figure**

**Page S5-S11**

**Fig. S1** Synthesis of rhMYDGF by PCR and identification by restriction enzymatic analysis. The strategy for synthesizing rhMYDGF is described in “Material and methods.” **a**: Schematic representation of rhMYDGF expression vector. **b**: Identification of recombinant plasmid by enzyme digestion (Xbal and Xhol). Lanes 1: pET31b-rhMYDGF-C‘-His; Lanes 2: pET-31b-rhMYDGF-C‘-His digested with Ndel-Xhol, the two bands represent pET-31b and rhMYDGF respectively. Lanes M: DNA marker.

**Fig. S2 a:** The gene sequence of expressed MYDGF; **b:** The amino acid sequence of MYDGF; **c:** The theoretical molecular weight of rhMYDGF predicted by swiss-prot;

**Fig. S3**: The results of rhMYDGF samples by CE-SDS analysis;

**Fig. S4 a**: The MYDGF spatial structure model predicted according to its amino acid sequence; **b**: The molecular weights analysis of DTT-treated protein and non-treated samples by SDS page.

**Fig. S5**: The results of rhMYDGF sample was detected by mass spectrometry analysis

**Fig. S6**: **a:** Time-response of change in HCAECs downstream MAPK and STAT3 signal of vehicle (PBS). **b**: Western blot analysis was performed to examine the protein levels of p-MAPK1/3, p-STAT3(S727) and p-STAT3(Y705) by image J (two independent sample t-test).

**Fig. S7:** The effect of rhMYDGF on PI3K signal pathway; **a:** Representative immunoblots showing phosphor (P)-AKT (T308), P-AKT (S473) and AKT expression in the presence or absence of LY294002 (20μM). **b:** Western blot analysis was performed to examine the protein levels of p-AKT (T308) and p-AKT (S473) by image J. *P < 0.05, **P < 0.01 and ***P < 0.001 versus baseline (two independent sample t-test); **c:** Proliferation activity analysis of HCAECs with different concentrations of rhMYDGF in the absence or presence of LY294002 (20μM) in vitro. *P < 0.05, **P < 0.01 and ***P < 0.001 versus control.

**Supplementary Figure**


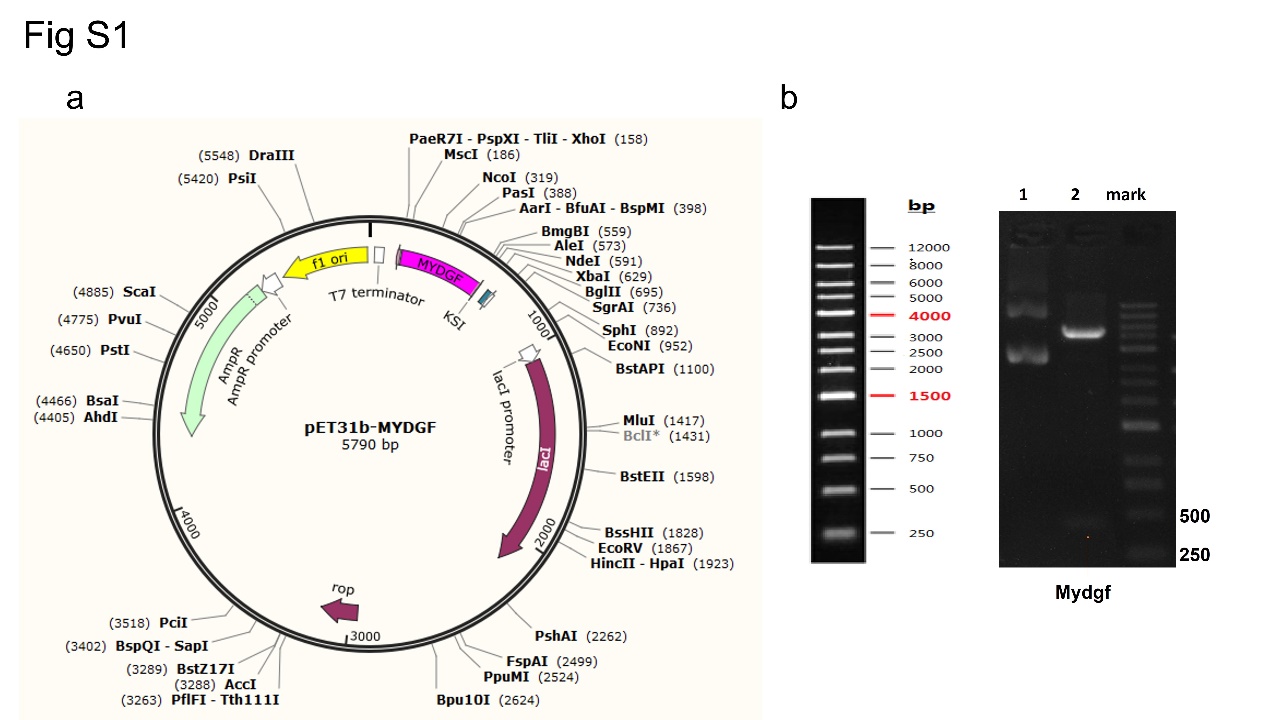


**Fig. S1** Synthesis of rhMYDGF by PCR and identification by restriction enzymatic analysis. The strategy for synthesizing rhMYDGF is described in “Material and methods.” **a**: Schematic representation of rhMYDGF expression vector. **b**: Identification of recombinant plasmid by enzyme digestion (Ndel and Xhol). Lanes 1: pET31b-rhMYDGF-C‘-His; Lanes 2: pET-31b-rhMYDGF-C‘-His digested with XhoI-XbaI, the two bands represent pET-31b and rhMYDGF respectively. Lanes M: DNA marker


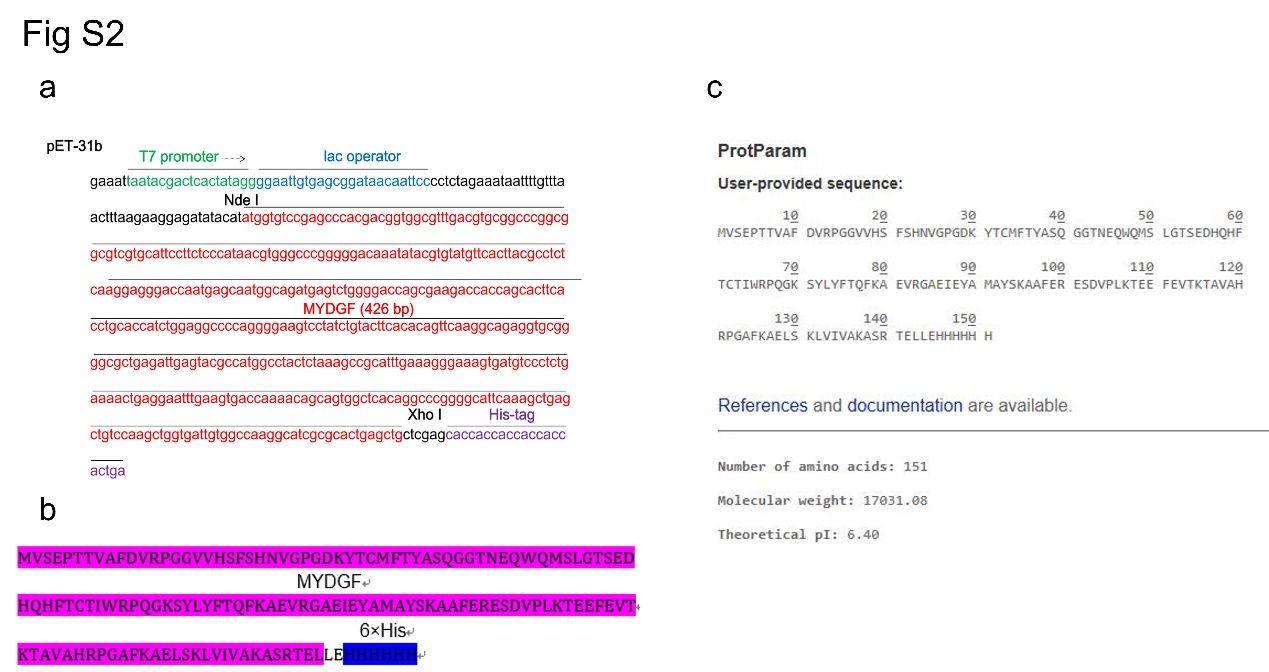


**Fig. S2 a:** The gene sequence of expressed MYDGF; **b:** The amino acid sequence of MYDGF; **c:** The theoretical molecular weight of rhMYDGF predicted by swiss-prot;


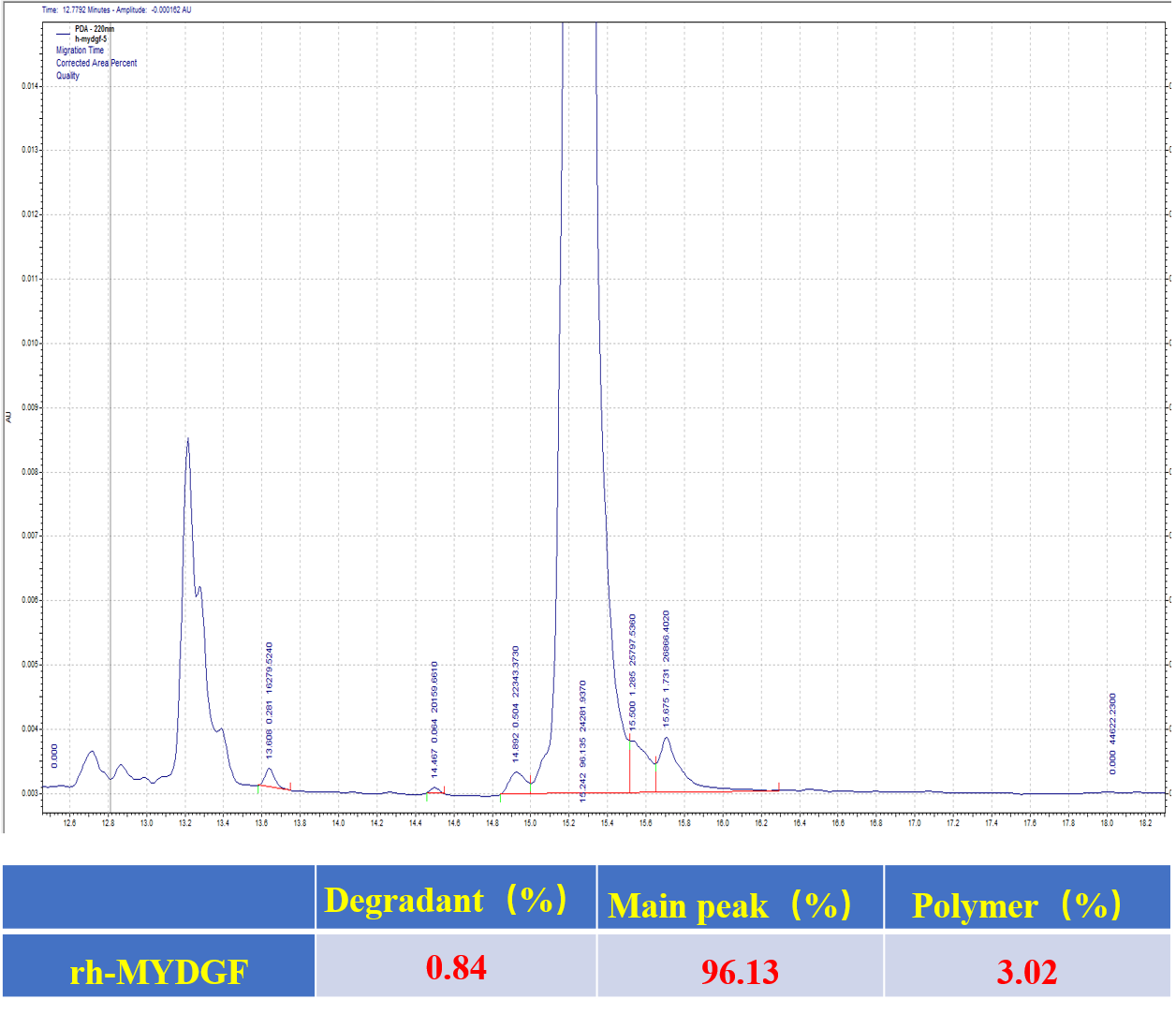


**Fig. S3** The results of rhMYDGF samples by CE-SDS analysis


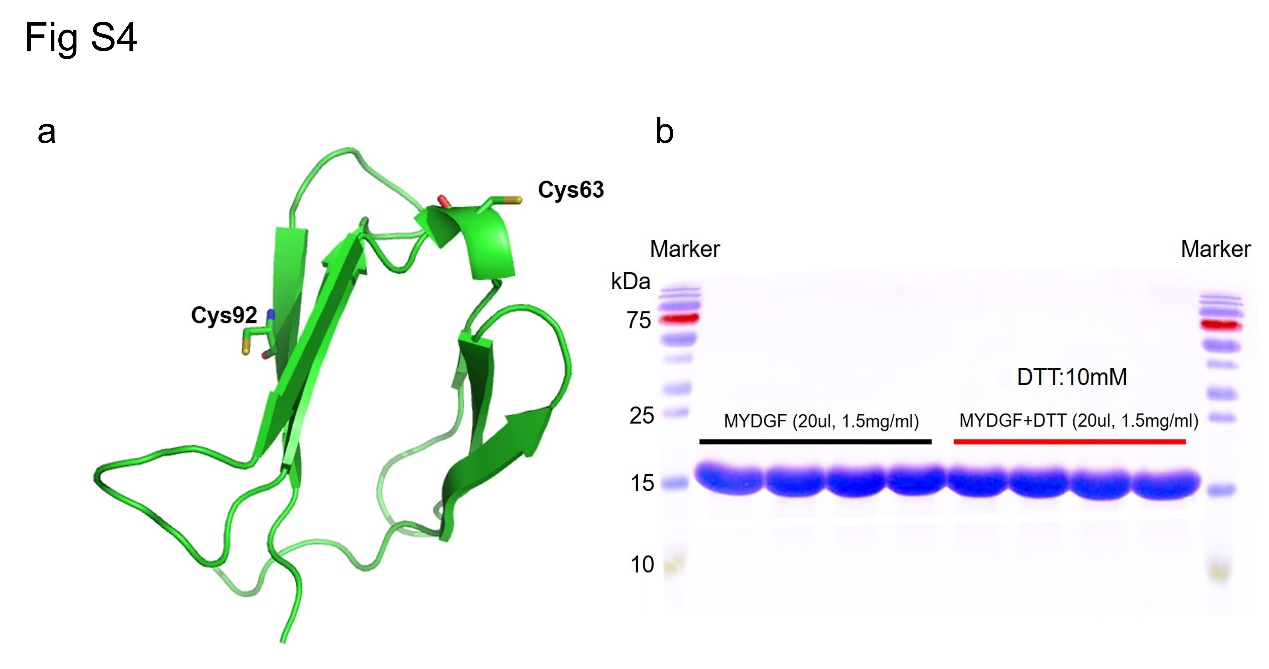


**Fig. S4 a**: The MYDGF spatial structure model predicted according to its amino acid sequence by the SWISS-MODEL (<https://swissmodel.expasy.org/>, a fully automated protein structure homology-modelling server, accessible via the ExPASy web server); **b**: The molecular weights analysis of DTT-treated protein and non-treated samples by SDS page.


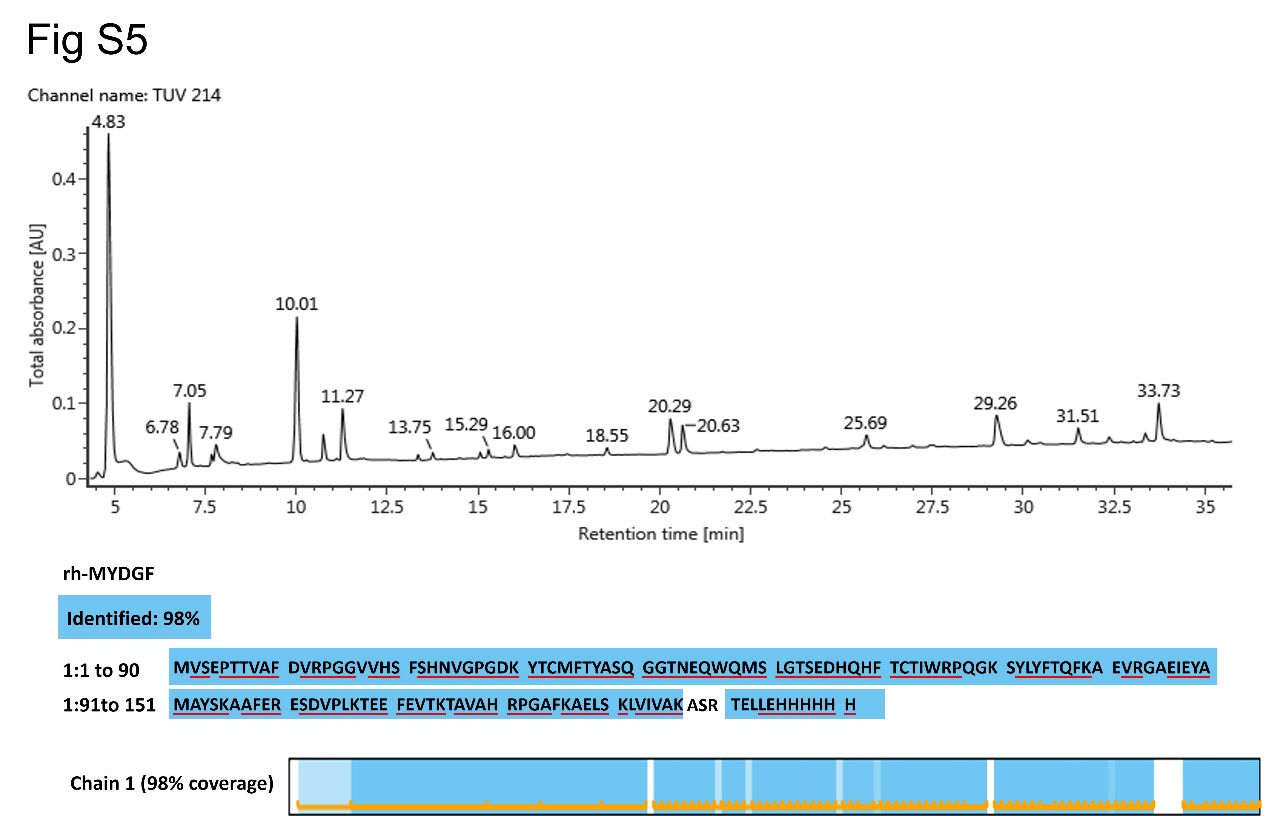


**Fig. S5** The results of rhMYDGF sample was detected by mass spectrometry analysis


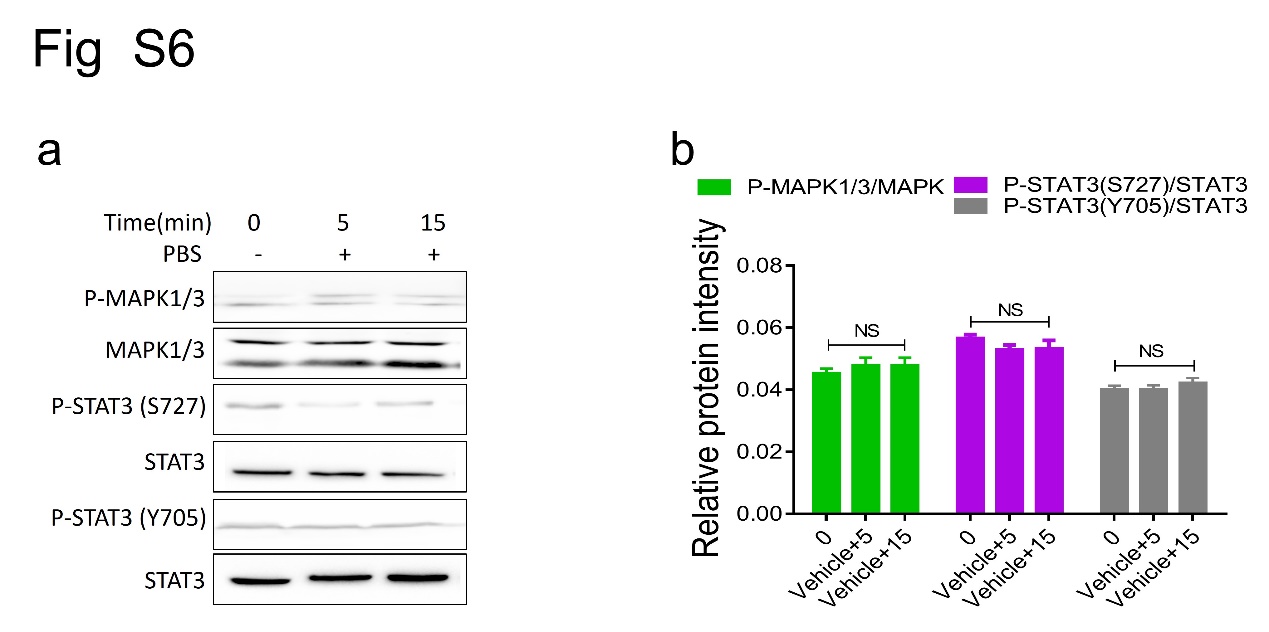


**Fig. S6**: **a:** Time-response of change in HCAECs downstream MAPK and STAT3 signal of vehicle (PBS). **b**: Western blot analysis was performed to examine the protein levels of p-MAPK1/3, p-STAT3(S727) and p-STAT3(Y705) by image J (two independent sample t-test).


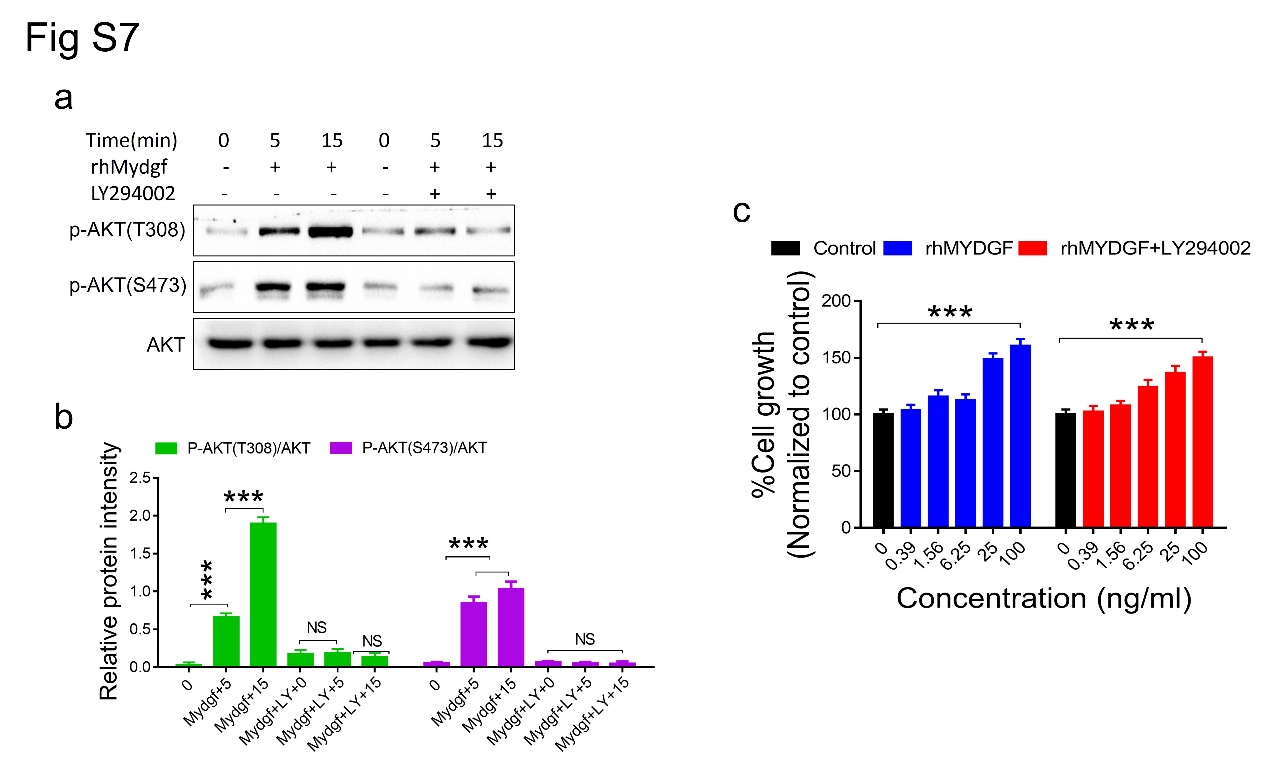


**Fig. S7:** The effect of rhMYDGF on PI3K signal pathway; **a:** Representative immunoblots showing phosphor (P)-AKT (T308), P-AKT (S473) and AKT expression in the presence or absence of LY294002 (20μM). **b:** Western blot analysis was performed to examine the protein levels of p-AKT (T308) and p-AKT (S473) by image J. *P < 0.05, **P < 0.01 and ***P < 0.001 versus baseline (two independent sample t-test); **c:** Proliferation activity analysis of HCAECs with different concentrations of rhMYDGF in the absence or presence of LY294002 (20μM) *in vitro*.
